# Supplementary material for: Genome-Wide Meta-Analysis of QTLs Associated with Root Traits and Implications for Maize Breeding
Source: Int J Mol Sci. 2023 Mar 24;24(7):6135. doi: 10.3390/ijms24076135 (PMC10093813; doi:10.3390/ijms24076135)
Supplement: Supplementary file 1 [file ijms-24-06135-s001.zip › Supplementary figures S1 & S2.pdf]

Deciphering key genomic regions associated with root traits and implications for maize breeding

Krishna Sai Karnatam[1]†, Gautam Chhabra[1]†, Dinesh Kumar Saini[2], Rajveer Singh[1], Gurwinder Kaur[1], Umesh Preethi Praba[1], Pankaj Kumar[1], Simran Goyal[1], Priti Sharma[1], Rumesh Ranjan[2], Surinder K Sandhu[2], Ramesh Kumar[3] and Yogesh Vikal[1*]

[1]School of agricultural Biotechnology, Punjab Agricultural University, Ludhiana-141001, India
[2]Department of Plant breeding and Genetics, Punjab Agricultural University, Ludhiana-141001, India
[3]Indian Institute of Maize Research, Ludhiana-141001, India

*Correspondence: Yogesh Vikal
Email Id: yvikal-soab@pau.edu

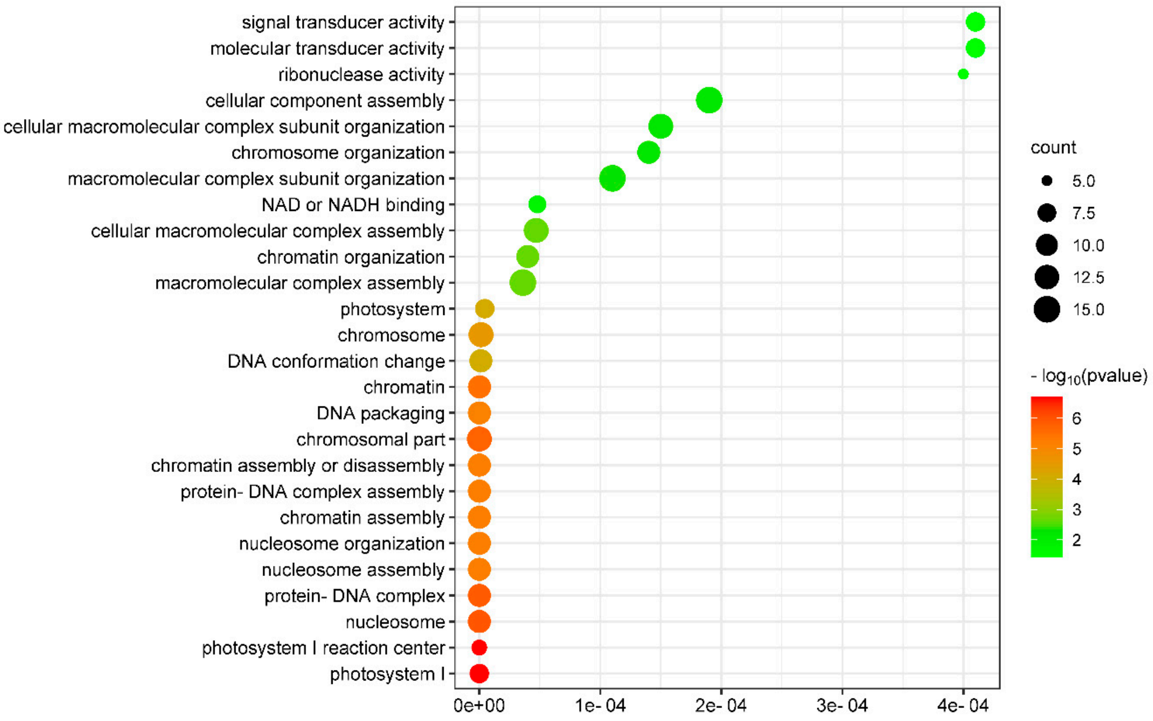

Figure S1. GO enriched in the genes in MQTLs associated with root traits in maize.

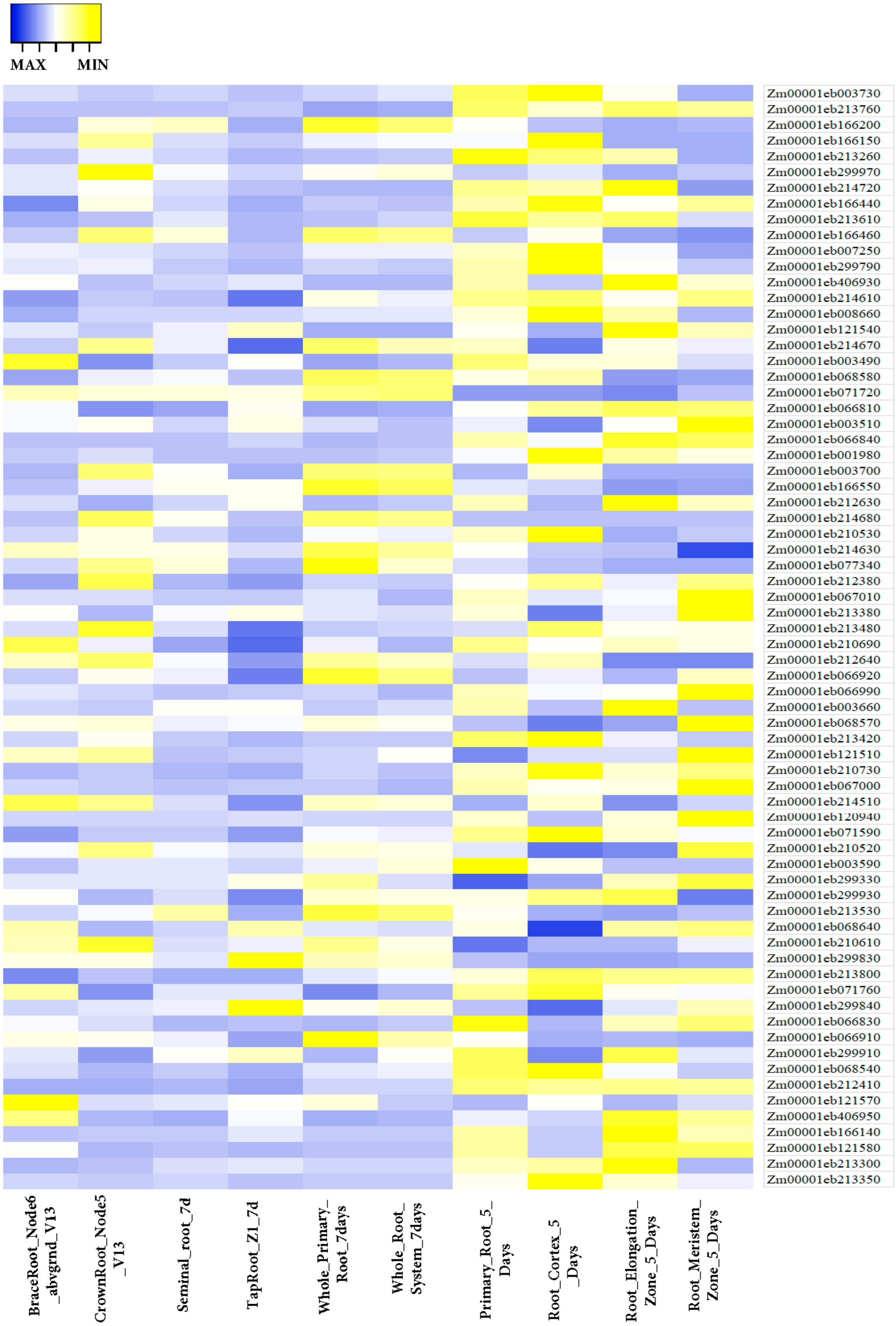

Figure S2. Expression patterns of selected 69 promising candidate genes in different root tissues. Heat map representing TPM value according to intensity of colour, blue (max TPM value) and yellow (min TPM value).
